# A 25 mm Circular Stapler Anastomosis Is Associated with Higher Anastomotic Leakage Rates Following Minimally Invasive Ivor Lewis Operation

**DOI:** 10.3390/jcm11237177

**Published:** 2022-12-02

**Authors:** Tobias Hofmann, Matthias Biebl, Sebastian Knitter, Uli Fehrenbach, Sascha Chopra, Candan Cetinkaya-Hosgor, Jonas Raakow, Philippa Seika, Rupert Langer, Johann Pratschke, Christian Denecke, Dino Kröll

**Affiliations:** 1 Department of Surgery, Campus Charité Mitte|Campus Virchow Klinikum, Charité—Universitätsmedizin Berlin, Corporate Member of Freie Universität Berlin and Humboldt-Universität zu Berlin and Berlin Institut of Health, Augustenburger Platz 1, 13353 Berlin, Germany; tobias.hofmann2@charite.de (T.H.); matthias.biebl@charite.de (M.B.); sebastian.knitter@charite.de (S.K.); sascha.chopra@charite.de (S.C.); cetinkaya.candan@gmail.com (C.C.-H.); jonas.raakow@charite.de (J.R.); philippa.seika@charite.de (P.S.); johann.pratschke@charite.de (J.P.); 2 Department of Radiology, Charité—Universitätsmedizin Berlin, Corporate Member of Freie Universität Berlin and Humboldt-Universität zu Berlin and Berlin Institut of Health, Augustenburger Platz 1, 13353 Berlin, Germany; uli.fehrenbach@charite.de; 3 Institute of Clinical Pathology and Molecular Pathology, Kepler University Hospital, Johannes Kepler University, Krankenhausstrasse 9, 4021 Linz, Austria; rupert.langer@kepleruniklinikum.at; 4 Department of Visceral Surgery and Medicine, Inselspital, Bern University Hospital, University of Bern, 3010 Bern, Switzerland

**Keywords:** minimally invasive oesophagectomy, Ivor Lewis, MIE, anastomotic leakage, circular stapler size

## Abstract

(1) Background: Minimally invasive oesophagectomy (MIE) with intrathoracic anastomosis is increasingly used in treating patients with oesophageal cancer. Anastomotic leakage (AL) remains a critical perioperative complication, despite recent advances in surgical techniques. It remains unclear to what extent the size of the circular stapler (CS), a 25 mm CS or a bigger CS, may affect the incidence of AL. This study aimed to evaluate whether the CS size in oesophagogastrostomy affects the postoperative AL rates and related morbidity in MIE. (2) Methods: We conducted a retrospective review of consecutive patients who had undergone thoracic MIE between August 2014 and July 2019 using a CS oesophagogastric anastomosis at the level of the Vena azygos. The patients were grouped according to CS size (mm): small-sized (SS25) and large-sized (LS29). The patient demographics, data regarding morbidity, and clinical outcomes were compared. The primary outcome measure was the AL rate related to the stapler size. (3) Results: A total of 119 patients were included (SS25: *n* = 65; LS29: *n* = 54). Except for the distribution of squamous cell carcinoma, the demographics were similar in each group. The AL rate was 3.7% in the LS29 group and 18.5% in the SS25 group (*p* = 0.01). The major morbidity (CD ≥ 3a) was significantly more frequent in the SS25 group compared with the LS29 group (*p* = 0.02). CS size, pulmonary complications, and cardiovascular disease were independent risk factors for AL in the multivariate analysis. (4) Conclusions: A 29 mm CS is associated with significantly improved surgical outcomes following standard MIE at the level of the azygos vein and should be conducted whenever technically feasible.

## 1. Introduction

Neoadjuvant treatment with oesophageal resection and lymphadenectomy with intrathoracic anastomosis is the standard technique in patients with oesophageal cancer [1]. Intrathoracic anastomosis has been performed more frequently in recent years than cervical anastomosis, with advantages in leakage and stricture rate [2].

Intrathoracic anastomosis is technically challenging, especially via the thoracoscopic approach. Intrathoracic anastomotic leakage (AL) after Ivor Lewis oesophagectomy is still considered the most serious postoperative complication, as it often results in prolonged hospitalization, higher postoperative mortality, and reduced long-term survival [3,4,5]. 

The ideal anastomotic technique remains controversial, with different variations (hand-sewn, linear stapled, and circular stapled) reported in the literature [6,7,8]. The widespread implementation of the circular stapler technique is often attributed to the intuitive handling associated with a lower AL rate than other techniques [9]. The most commonly used diameter of the circular stapler for oesophageal reconstruction is either 25 mm using an orally inserted anvil or via thoracoscopy, or a larger diameter (28 to 29 mm), which have been increasingly used lately [10,11,12]. 

Numerous published studies have investigated the effect of anastomotic techniques and the stapler diameter on the subsequent stricture rates after oesophagectomy. According to recent meta-analyses, using a larger circular stapler was associated with an increased risk of anastomotic strictures [13]. However, data on the impact of the CS size and associated leakage rates in MIE with circular stapled anastomoses are scarce and controversial [10,11]. 

Therefore, our study aimed to review our institution’s experience regarding the safety and efficacy of minimally invasive thoracic oesophagogastric end-to-side anastomosis performed using two different CS diameters.

## 2. Methods

### 2.1. Study Design

This retrospective study aimed to evaluate whether different sizes of circular endoscopic stapling devices delivering two rows of titanium staples would impact the AL rate of intrathoracic stapled circular anastomoses during elective oesophageal cancer resection.

According to the circular stapler (CS) size, the evaluated patient cohort was divided into a small-sized group (25 mm diameter stapler; SS25 group) and a large-sized group (29 mm diameter stapler; LS29 group). Subgroup analysis was performed to differentiate between intrathoracic OrVil double-stapling (*n* = 40) vs. the purse-string anastomosis technique (*n* = 25) in the SS25 group.

The primary study outcome parameter was the occurrence of anastomotic leakage within the hospital stay after minimally invasive Ivor Lewis resection.

AL was graded according to definitions stated by the Oesophagectomy Complications Consensus Group (ECCG) [14]. Secondary study outcome parameters included postoperative all-cause mortality (in-hospital, 30 days and 90 days) and postoperative morbidity as defined by the Clavien–Dindo (CD) classification [15] and according to the ECCG definitions. ALs were assessed using computed tomography (CT) scans of the thorax and abdomen with oral contrast fluid or a gastroscopy. Anastomotic stricture was defined as dysphagia requiring endoscopic treatment within three months.

### 2.2. Patients

We conducted a retrospective study of all consecutive patients who underwent oesophageal resection for cancer of the intrathoracic oesophagus or gastroesophageal junction (Siewert type I and type II) between 08/2014 and 07/2019 at the Department of Surgery, Charité University Medicine Berlin (*n* = 327).

Patients were included in the analysis if the oesophagectomy was performed including a minimally invasive Ivor Lewis resection comprising total minimally invasive, minimally hybrid invasive (abdomen open), and robotic resections. Patients were included in the analysis if a 25 or 29 mm CS was used for the esophagogastric intrathoracic anastomosis.

The following patients were excluded from analysis: Patients who underwent open thoracic resection, non-curatively intended or emergency resection, surgery for indications other than oesophageal adenocarcinoma or squamous cell cancer, patients < 18 years of age, patients who had received a cervical anastomosis, reconstruction other than a gastric pull-up or no reconstruction at all, patients who underwent a two-stage reconstruction, and patients who received an anastomosis other than a 2-row circular stapled anastomosis (hand-sewn, linear stapled).

Next, the postoperative CT scans of all the remaining patients were obtained and reviewed by a consultant radiologist blinded to the type of stapler used. Care was taken to include only patients receiving a standard Ivor Lewis resection and reconstruction with a circular end-to-side oesophagogastrostomy at the level of the vena azygos (defined as within a 2 cm range above or below the stump of the transected Vena azygos following postoperative contrast-enhanced CT scan to rule out sampling bias. This measure was necessary because a large circular stapler often cannot be accommodated by the oesophagus in high intrathoracic anastomoses. Consequently, patients with no available postoperative CT scan were excluded from further analysis.

**Figure 1:**
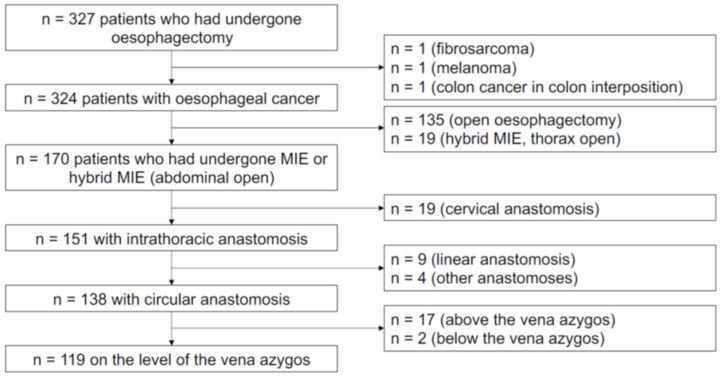
Study inclusion and exclusion criteria flow chart.

In total, 119 patients met the inclusion criteria and had postoperative CT scans available for review. The full selection process is depicted in Figure 1. All patients provided written informed consent for the procedure. Data collection and the retrospective study were performed under the approval of the Charité institutional review board (IRB).

### 2.3. Outcome Measures and Definitions

The following data were collected from our institutional prospectively maintained electronic database and electronic patient charts: patient demographics and related comorbidities, tumour-specific variables, use of neoadjuvant treatment, and perioperative and postoperative data up to 90 days post-surgery. Tumours were classified according to the World Health Organization classification; staging was performed according to the Union for International Cancer Control (UICC)/American Joint Committee on Cancer (eight editions) criteria [16].

### 2.4. Surgical Techniques

The patients underwent a standard Ivor Lewis oesophagectomy with a two-field lymphadenectomy and a minimally invasive intrathoracic circular stapled anastomosis via a transabdominal (laparoscopic or open) and right thoracoscopic approach. A 25 mm or 29 mm diameter 2-row circular stapler was used for anastomoses.

The abdominal part comprised of a median laparotomy or five-port laparoscopy with a standard D2- lymphadenectomy around the branches of the celiac trunk, gastric cardia, and lower mediastinum, and gastrolysis with careful preservation of the right gastro-omental pedicle utilizing a partial Kocher manoeuvre. The abdominal part was performed open or laparoscopically/robotically using a similar technique. Reasons for open dissection were an additional abdominal organ resection, a status of post extensive previous abdominal surgery and adhesions, or a demand for D3 lymphadenectomy.

Under preservation of the right gastric vessels, a partial division of the stomach from the lesser curvature starting approximately 6 cm proximal to the pyloric ring was performed to create a 5 to 6 cm wide gastric tube, which was later completed during the thoracic phase through the retrieval incision using a laparoscopic linear stapler with green and blue cartridges. After careful dissection of the anteriorly widened oesophageal hiatus, the abdominal phase was completed. A silicone drain was left at the hiatus in the case of open dissection, while minimally invasive procedures were not routinely drained.

Next, the patient was repositioned in a left lateral position to perform a four-port video-assisted thoracic dissection. After achieving stable left-sided single-lung ventilation, the thorax was entered through a 6 cm mini-thoracotomy at the level of the posterior axillary line in the fourth intercostal space, and an Alexis foil self-retaining ring (Applied Medical, Rancho Santa Margarita, CA, USA) was inserted. A 12 mm trocar was introduced in the 6th intercostal space (ICS), and two 12 mm trocars were introduced in the 9th ICS. For robotic access, the trocars were positioned at 4th, 6th, 8th, and 10thICS along the posterior axillary line, together with one 12 mm assist trocar in the 7th ICS. The inferior pulmonary ligament was divided, and the oesophagus was mobilised to visualise the mesoesophagus, which was then transected close to the aorta. Next, the oesophagus was looped with a silicone drain for retraction and circular dissection. The azygous vein was routinely divided with an endoscopic stapler, or secured by two locking clips on either side and transected. Mediastinal compartment lymphadenectomy along the oesophagus, carinal region, and around the azygos vein was routinely performed. The intended transection level of the oesophagus was confirmed by intraoperative endoscopy, and the oesophagus was divided with a linear endoscopic stapler. The gastric tube was brought into the thorax, and the specimen was retrieved using right-sided minithoracotomy. This was the same for the robotic-assisted MIE and the laparoscopic MIE. Outside the thorax, the gastric tube was completed, and the specimen was removed. After gross back table inspection, a frozen section examination of the proximal margin was performed.

#### 2.4.1. Anastomotic Techniques

Two anastomotic techniques were applied. For an OrVil double-stapling anastomosis (only for 25 mm stapling), after circumferential liberation of the oesophageal stump, the staple line was aligned using two endoscopic clamps, and the 25 mm anvil was placed by transoral insertion using a 90 cm PVC tube, which was brought out precisely in the middle of the linear oesophageal staple line. 

In the case of a purse-string anastomosis, the staple line was cut from the oesophagus, and a 2-0 Prolene running suture was placed around the oesophageal cut-surface. After insertion of the anvil, the suture was firmly closed, and a second 2-0 Prolene purse-string suture was used to secure the insertion site. The anvil-corresponding 25 or 29 mm (ILS; 3.5 mm, Ethicon, Cincinnati OH, USA) circular stapler was inserted by terminal incision of the gastric tube through the retrieval incision in the 4th ICR. With retrograde intubation of the stomach, the spike was brought out on the posterior (mesenteric) wall of the gastric tube.

Following completion of the end-to-side anastomosis, the stapler was removed, and the gastric incision was closed using a linear endoscopic stapler. Finally, an omental wrap was used to encircle the anastomosis. Repeat endoscopy using air-leak testing confirmed the primary integrity of the anastomosis, and a nasogastric tube was placed for 48 h. No final control of blood perfusion using indocyanine green fluorescence was used during the study period. After removing all ports, a chest tube was placed in the thoracic cavity, and the mini-thoracotomy was closed.

#### 2.4.2. Postoperative Management

After the operation, patients were transferred to the intensive care unit (ICU) for respiratory and hemodynamic monitoring. On the first postoperative day, hemodynamically and stable respiratory patients were transferred to the surgical ward. Subsequently, between the first and second postoperative day, patients started with sips of water, which was given free ad libitum on the third postoperative day. On the 4th day postoperatively, patients started enteral feeding with liquid food provided there were no clinical signs of anastomotic leakage. Hereafter, oral intake was gradually increased to solid food. Oesophageal swallow tests to prove or exclude anastomotic leakage were not routinely performed. There was no enhanced recovery or fast-track program during the study period.

### 2.5. Statistical Analysis

Quantitative and qualitative variables were expressed as medians (range) and frequency. Chi-squared or Fisher’s exact test for categorical variables and the Mann–Whitney U test for continuous variables were used as appropriate tests to compare the groups. Comparisons between the survival rates were performed using log-rank tests. To identify factors associated with AL after MIE, the following clinical and pathological variables were subjected to univariate analysis: male sex, age > 65 years, BMI > 30 kg/m^2^, tumour location, diabetes, cardiovascular disease, pulmonary disease, renal insufficiency, preoperative chemotherapy, preoperative radiotherapy, histologic type, staple diameter, pulmonary complications, and postoperative pneumonia. Multivariable analyses were calculated with binary logistic regression using the stepwise backward conditional model. In this model, significant variables from the univariate analysis as well as clinically relevant variables were included. In the subsequent multivariate analysis, all factors with a *p*-value < 0.1 were entered in a logistic regression model. Backward stepwise (Wald) regression analyses were performed, for parameter selection. Three independent variables (small stapler diameter, pre-existing cardiovascular conditions, and perioperative pulmonary complications) were included in the final multivariate logistic regression model. Model fit was evaluated using Nagelkerke pseudo R-squared. *p*-values < 0.05 were considered statistically significant. Statistical analysis was performed using the SPSS software package, version 26 (IBM, Armonk, NY, USA). 

## 3. Results 

### 3.1. Baseline Characteristics

In total, 119 patients were included in the study (SS: *n* = 65; LS: *n* = 54). The baseline demographics and clinical characteristics are shown in Table 1. More squamous cell carcinomas were seen in the SS25 group (*n* = 20; (31.7%) vs. the LS29 group, *n* = 8; (15.4%), *p* = 0.042). No significant differences were found in age, sex, body mass index (BMI), comorbidities, ASA classification, tumour localization, preoperative therapy, or recorded UICC stage.

### 3.2. Morbidity and Mortality

The perioperative outcomes are summarised in Table 2. Robotic surgery was performed in 22 (18.5%) of the cases. Overall, anastomotic leakage was observed in 14 patients (11.7%). The incidence of anastomotic leakage was significantly lower in the LS29 group than in the SS25 group (SS, *n* = 12 (18.5%) vs. LS, *n* = 2 (3.7%); *p* = 0.013)). Major anastomotic failure/morbidity (CD ≥ 3a) was significantly more frequent in the SS25 group compared to LS 29 group (*p* = 0.02). No significant differences were found in the surgery duration, postoperative pulmonary complications, mortality, time of hospitalization, or overall survival between the groups. The incidence of anastomotic stricture was not significantly different between the SS25 and LS 29 groups postoperatively. 

### 3.3. Univariate Analysis/Multivariate Analysis

The results of univariate and multivariate analyses of risk factors for anastomotic leakage are summarised in Table 3. Upon univariate analysis, pneumonia, pulmonary complications, and a small stapler diameter were significant risk factors for AL. In multivariate analysis, small stapler diameter (OR: 2.435; 95% CI = 1.093–5.426; *p* = 0.029) and pulmonary complications (OR: 0.360; 95% CI = 0.163–0.796; *p* = 0.012) were identified as independent risk factors for anastomotic leakage.

### 3.4. Subgroup Analysis

Analysis of SS25 group patients using an intrathoracic OrVil double-stapling (*n* = 40) vs. purse-string anastomosis technique (*n* = 25) showed no significant differences regarding AL, anastomotic stricture, major postoperative morbidity, or pulmonary complications. The AL rate after double stapling was 17.5%, and end-to-side purse-string was 20.0% (*p* = 0.065).

## 4. Discussion

Circular anastomosis staplers are frequently used to construct minimally invasive intrathoracic oesophagogastric anastomoses after Ivor Lewis esophagectomy because of their accessible and straightforward use [17]. Currently, there is no evidence-based recommendation regarding the appropriate size of the circular stapler for esophagogastric anastomoses, both in terms of anastomotic leakage and postoperative outcome, especially in MIE.

In this study we demonstrated that the incidence of anastomotic leakage after oesophagectomy was significantly higher when a small-sized CS (25 mm) was used compared to a large-sized CS (29 mm) (SS, *n* = 12 (18.5%) vs. LS, *n* = 2 (3.7%); *p* = 0.013).

The incidence of overall AL (11.8%) was consistent with rates published by the ECCG [14] and was favourable when compared to other minimally invasive studies [6,18].

Müller and co-workers recently published their single-centre experience of 632 patients who had undergone transthoracic stapler anastomosis using either a 25 mm or a 28 mm circular stapler [11]. They had a nonsignificant trend towards a lower leakage rate in the 25 mm cohort (15.4%) compared to the 28 mm cohort (10.8%). Notably, the authors predominantly found evidence that surgeons performed an open thoracic anastomosis, with rates of 71% (25 mm group) and 88% (28 mm group). Furthermore, the stapler size was chosen according to the patient’s anatomy with no specific level of anastomosis mentioned, likely contributing to a sampling bias. 

In our cohort, we reviewed the outcome of CS anastomosis with the exact stapler diameter of the anastomosis performed at the vena azygos level, and all patients with a higher anastomosis were excluded. This was done for two reasons; firstly, a larger stapler is not feasible for an extremely high intrathoracic anastomoses due to the small size of the oesophageal lumen. Secondly, the incidence of anastomotic leaks may be increased in very high intrathoracic anastomosis resulting in bias. While there is no conclusive literature, open thoracotomy may allow for easier handling of a large size stapler than minimally invasive surgery; therefore, we excluded patients with an open transthoracic approach. However, we restored the continuity through a right-mini-thoracotomy using a 29 mm stapler for anastomosis in our minimally invasive group.

Tagkalos et al. found no differences between the use of a smaller (25 mm) and larger size (28 mm) of the circular stapler regarding the incidence of AL [10]. The authors, however, included open and hybrid approaches in the analysis, and again the level of the anastomosis in the oesophagus was not reported. 

In contrast, our cohort showed that a small-sized CS (25 mm) was identified as a risk factor for anastomotic leakage on univariate and multivariate analysis. 

Anastomotic leakage may be influenced by the location, side, and type, and by the calibre of the anastomosis. When using a circular stapler, there is no difference in stapled height between stapler sizes, preventing blood loss and leaks that can occur with a linear stapler due to a mismatch between the stapler height and the tissue thickness (1.5–2.2 mm in closed stapled height according to the published staple height information of the stapler manufacturer).

One of the specific issues in oesophageal surgery is the composition of the oesophageal wall. Unlike other parts of the gastrointestinal tract, the oesophagus’ muscular layer is prone to hypertrophy. In a typical western population, many patients with oesophageal cancer present with decompensated stenosis and dysphagia due to tumour growth. As the anastomosis to the gastric sleeve is usually performed in end-to-side, a purse-string closure of the oesophagus accommodating the circular stapler anvil is required. With an enlarged diameter of the oesophagus and a thickened wall, a 25 mm anvil does not accommodate all layers of the oesophageal wall. A larger stapler allows more mucosa and subserosa to be placed in the space between the stapler and the anvil inside the staple line. The surface of the anastomosis is increased, decreasing the tension and the risk of leakage. 

When using a 29 mm anvil, the doughnuts created during the anastomosis firing are significantly thicker, guaranteeing that the anastomosis does not cut through the muscular layer of the oesophagus. Our findings, together with those of Mueller et al. [11], suggested that this mechanically logical explanation may be part of the pathophysiology of anastomotic leakage generation following an oesophagogastrostomy in an Ivor Lewis procedure. There is, however, limited literature that has explored this mechanism in detail. 

Even more, double stapling techniques results in two “dog-ears,” harbouring the risk of malperfused areas next to the stapled anastomosis, which may result in leakages. The multicentre analysis of Schröder et al. [6] revealed a markedly increased leakage rate after intrathoracic end-to-side double stapling technique (23.3%) compared to end-to-side purse-string (13.9%) oesophagogastrostomies.

Notably, the outcome in our small subanalysis cohort of patients receiving a double-stapled 25 mm anastomosis did not significantly differ from the 25 mm purse-string cohort related to AL. It was comparable to the higher AL rates of the studies mentioned above. However, we cannot rule out a type II statistical error due to the limited sample size of our cohort. 

Regarding postoperative stricture formation, a literature search on the current techniques and approaches for intrathoracic anastomosis was summarized in 2012 by Mass et al. [19]. Twelve studies were evaluated for anastomotic leakage and stenosis rates. The review found no essential differences between the transoral and transthoracic use of staplers. These results contrast a scientific review showing that the use of larger circular stapler sizes was strongly associated with a reduced risk of strictures after oesophagectomy [13]. Results from our study do not support the conclusion of Allen et al., as there were no differences in the stricture rates between the two sizes of circular stapler diameter used for oesophagogastric anastomosis.

In addition to stapler size, we found pulmonary complications to be a significant risk factor for AL on multivariate analysis. While several authors also report an association between pneumonia and AL, it is often described as a consequence rather than a pre-existing condition. However, the relationship between these, often concomitant, complications is complex. Infection induces an inflammatory response at the anastomotic site, which inhibits collagen deposition, impeding wound healing and resulting in a sustained connection between the intraluminal and extraluminal microbiome. This facilitates bacterial translocation to the thoracic cavity, sustaining the infection and slowing the anastomosis recovery further.

The limitations of this study are its single-centre design, limited sample size, and retrospective design. A time bias for the 25 mm anastomosis, which was conducted more frequently in the beginning of the study period and no objective measured diameter of the oesophagus are further limitations. However, no significant difference in the incidence of AL was seen before 2017 vs. after 2017 in our study cohort. 

While our study comprised only minimally invasive intrathoracic anastomoses, other studies analysed unselected patient cohorts including all types of (open, hybrid, and minimally invasive) esophagectomies and various reconstruction techniques.

The size of the stapler at the vena azygos level was chosen based on intraoperative assessment of the circumference of the oesophageal lumen to create an optimal esophagogastric anastomosis, using the largest possible circular stapler size where appropriate. However, patients with squamous cell carcinoma more often underwent a 25 mm anastomosis, which can be attributed to anatomical reasons (e.g., smaller oesophageal diameter after neoadjuvant radiotherapy). Overall, the choice of the circular stapler size should be adapted to the patient´s anatomy, tissue quality (neoadjuvant radiotherapy), diameter of the oesophagus, and other patient-associated risk factors. Novel circular stapler designs (e.g., tristapler, elliptical angled interface), or the use of intraoperative angiography using indocyanine green (31), are promising but require further testing in prospective studies. 

## 5. Conclusions

This single-centre cohort study demonstrated that the incidence of AL and related major morbidity after MIE was significantly lower using the large-sized (29 mm diameter) circular stapler than using a 25 mm diameter stapler for minimally invasive end-to-side oesophagogastrostomies performed at the level of the azygos vein. Therefore, we advocate using a large-diameter stapler whenever possible for standard minimally invasive Ivor Lewis resections.

## Figures and Tables

**Table 1:** Baseline demographics and clinical characteristics.

Characteristic	SS (*n* = 65)	LS (*n* = 54)	*p*
Male sex, *n* (%)	50 (76.9)	49 (88.9)	0.088
Median age at resection, years (range)	63.5 (39–84)	65.8 (44–81)	0.239
Median BMI, kg/m^2^ (range)	28.0 (18–43)	26.3 (16-51)	0.235
Tumour location, *n* (%)			0.470
Oesophagus	33 (50.8)	31 (57.4)	
GEJ	32 (49.2)	23 (42.6)	
Comorbidities			
Diabetes, *n* (%)	10 (15.4)	6 (11.1)	0.496
Cardiovascular disease, *n* (%)	39 (52.7)	37 (64.8)	0.59
Pulmonary disease, *n* (%)	13 (20.0)	9 (16.7)	0.641
Renal insufficiency, *n* (%)	6 (9.2)	5 (9.3)	0.996
ASA physical status, *n* (%)			0.497
I	1 (1.6)	2 (4.0)	
II	26 (41.3)	16 (32.0)	
III	35 (55.6)	32 (64.0)	
IV	1 (1.6)	0 (0.0)	
Preoperative chemotherapy, *n* (%)	55 (84.6)	50 (92.6)	0.179
Preoperative radiotherapy, *n* (%)	23 (35.9)	16 (30.2)	0.511
UICC stage, *n* (%)			0.773
I	7 (11.5)	3 (6.1)	
II	16 (26.2)	12 (24.5)	
III	35 (57.4)	31 (63.3)	
IV	3 (4.9)	3 (6.1)	
Histologic type, *n* (%)			**0.042**
AC	43 (68.3)	44 (84.6)	
SCC	20 (31.7)	8 (15.4)	

BMI, body mass index; GEJ, gastroesophageal junction; ASA, American Society of Anaesthesiologists; UICC, Union for International Cancer Control; AC, adenocarcinoma; SCC, squamous cell carcinoma, SS small-sized circular stapler group, LS, large-sized circular stapler group. Statistical significance is indicated in bold.

**Table 2:** Operative and postoperative patient outcomes.

Characteristic	SS (*n* = 65)	LS (*n* = 54)	*p*
Median duration of resection (range), min	442.5 (306–631)	429.7 (254–561)	0.357
Median duration of hospital stay (range), days	26.6 (10–103)	22.9 (9–261)	0.477
Postoperative morbidity, *n* (%)	49 (75.4)	35 (67.3)	0.335
Major postoperative morbidity, *n* (%)	41 (63.1)	19 (35.2)	**0.002**
Anastomotic leak, *n* (%)	12 (18.5)	2 (3.7)	**0.013**
Anastomotic stricture within 90 days, *n* (%)	1 (1.6%)	4 (7.4.3)	0.175
Pyloric stenosis, *n* (%)	18 (27.7)	10 (18.9)	0.262
Pulmonary complications, *n* (%)	33 (50.8)	23 (42.6)	0.374
Postoperative pneumonia, *n* (%)	23 (35.4)	14 (25.9)	0.267
30-day mortality, *n* (%)	1 (1.6)	0 (0)	1
90-day mortality, *n* (%)	4 (7.3)	1 (2.9)	0.389

Statistical significance is indicated in bold.

**Table 3:** Risk factors for perioperative anastomotic leakage.

Characteristic	AL (*n* = 14)	UV *p*	MV †	
			*p*	HR (95% CI)
Male sex, *n* (%)	12 (85.7)	0.726		
Age > 65 years, *n* (%)	7 (50.0)	0.920		
BMI > 30 kg/m^2^, *n* (%)	4 (28.6)	0.343		
Tumour location, *n* (%)		0.157		
Oesophagus	5 (35.7)			
GEJ	9 (64.3)			
Diabetes, *n* (%)	3 (21.4)	0.358		
Cardiovascular disease, *n* (%)	12 (85.7)	0.071	0.070	0.474 (0.211–1.064)
Pulmonary disease, *n* (%)	5 (35.7)	0.087		
Renal insufficiency, *n* (%)	3 (21.4)	0.110		
Preoperative chemotherapy, *n* (%)	13 (92.9)	0.573		
Preoperative radiotherapy, *n* (%)	5 (35.7)	0.840		
Histologic type, *n* (%)		0.568		
AC	11 (79)			
SCC	3 (21)			
Stapler diameter, *n* (%)		**0.025**	**0.029**	2.435 (1.093–5.426)
25 mm	9 (69.2)			
29 mm	4 (30.8)			
Pulmonary complications, *n* (%)	12 (85.7)	**0.007**	**0.012**	0.360 (0.163–0.796)
Pneumonia, *n* (%)	9 (64.3)	**0.008**		

† Backward stepwise (Wald) Logistic regression multivariate analysis. Nagelkerkes R-squared = 0.308. UV, Univariate; MV, Multivariate; CI, confidence interval; HR, hazard ratio; BMI, body mass index; GEJ, gastroesophageal junction; AC, adenocarcinoma; SCC, squamous cell carcinoma. Statistical significance is indicated in bold.

## Data Availability

Data available from authors upon request.
